# Account of Diseases in an Eastern District of London, from the 20th of February to the 20th of March

**Published:** 1799-04

**Authors:** 


### *Account of Diseases in an Eastern District of London, from the 20th of February to the 20th of March.*

ACUTE DISEASES.

| | No. of Cases. |
|---|---|
| Typhus Gravior | 4 |
| Typhus Mitior | 3 |
| Peripneumonia Notha | 7 |
| Hectica | 2 |
| Ophthalmia | 1 |
| Acute Rheumatism | 3 |

CHRONIC DISEASES.

| | No. of Cases. |
|---|---|
| Cough | 17 |
| Dyspnœa | 12 |
| Cough and Dyspnœa | 10 |
| Hoarseness | 4 |
| Hæmoptoe | 2 |
| Phthisis Pulmonalis | 3 |
| Anasarca | 3 |
| Ascites | 2 |
| Hydrothorax | 2 |
| Cephalalgia | 7 |
| Vertigo | 3 |
| Epilepsia | 1 |
| Ophthalmia | 5 |
| Gastrodynia | 3 |
| Enterodynia | 5 |
| Diarrhœa | 30 |
| Diarrhœa biliosa | 12 |
| Lienteria | 3 |
| Dysenteria | 2 |
| Amenorrhœa | 5 |
| Fluor albus | 6 |
| Chlorosis | 3 |
| Cancer in utero | 1 |

Enu-

| | No. of Caſes. |
|---|---|
| Enureſis | 2 |
| Dyſuria | 5 |
| Hæmorrhois | 4 |
| Hernia | 2 |
| Tremor | 1 |
| Hyſteria | 3 |
| Hypochondriaſis | 4 |
| Chronic Rheumatiſm | 15 |
| Lumbago | 3 |
| Sciatica | 2 |

PUERPEREAL DISEASES.

| | No. of Caſes. |
|---|---|
| Ephemera | 2 |
| Menorrhagia Lochialis | 3 |
| Maſtrodynia | 4 |
| Rhagas Papillæ | 3 |

INFANTILE DISEASES.

| | |
|---|---|
| Ophthalmia | 4 |
| Ophthalmia purulenta | 3 |
| Tinea | 2 |

Since the laſt report, diſeaſes of the pneumonic kind, both of the acute and chronic ſpecies, have been leſs frequent than in the former months; the return of eaſterly and north-eaſterly winds has, however, prolonged the duration of the chronic ſpecies of theſe diſeaſes.

Diſorders of the bowels have been very frequent, and, in ſome inſtances, very tedious and obſtinate. They have appeared in various forms, but principally in that of *diarrhoea.* The periſtaltic motion of the inteſtinal canal has been increaſed to a morbid degree, and very frequent diſcharges of the contents of the bowels have taken place. Theſe diſcharges have been different in their quantity and conſiſtence, and attended with a variety of ſymptoms in different patients. Flatulence and borborygmi have preceded them in ſome inſtances, whilſt in others, they have been preceded, and accompanied with a conſiderable degree of pain. Teneſmus and ſmall diſcharges of a bloody and mucous appearance occurred, in ſome caſes, towards the cloſe of the diſeaſe. Theſe ſymptoms, however, occurred without any primary fever, and the diſeaſe might be diſtinguiſhed from dyſentery by this circumſtance, as well as by the diſcharge of fæces, which ſeldom takes place in the latter diſeaſe. In ſome of the inſtances referred to in the liſt, the diſcharges were ſo frequent and haſty, that aliment paſſed through the inteſtines in an undigeſted form, which circumſtance ſerved to characteriſe that ſpecies of the diſeaſe, called *lienteria.*

Some of the patients were affected by nauſea and vomiting, and moſt of them experienced a reduction of ſtrength, and a conſiderable degree of emaciation.

This diſeaſe, when confined within due bounds, often proves beneficial to the conſtitution, and if not checked in too early a ſtage, will generally terminate very favourably; but the early uſe of aſtringents, to ſtop the diſcharge, of opiates to relieve pain, or of warm and ſtimulating remedies to ſupport the ſtrength of the patient, are always injurious, and ſometimes produce the moſt fatal ſymptoms.

